# Evaluation of a phage-inclusive multimodal strategy for recurrent urinary tract infections

**DOI:** 10.1186/s12866-026-05524-4

**Published:** 2026-08-12

**Authors:** Simone C. Lieberknecht-Jouy, Thomas Fließwasser, Dajana Pavlovic, Lucas J. Fein, Verena Wiemann, Trang Do, Denia Frank, Tobias Weirauch, Annika Y. Classen, Silvia Würstle, Thomas A. Wichelhaus, Maria J. G. T. Vehreschild

**Affiliations:** 1 Department of Internal Medicine, Infectious Diseases, Goethe University Frankfurt, University Medicine Frankfurt, Frankfurt am Main, Germany; 2 Institute for Pharmaceutical Microbiology, University Hospital Bonn, University of Bonn, Bonn, Germany; 3 Institute of Medical Microbiology and Infection Control, Goethe University Frankfurt, University Hospital, Frankfurt am Main, Germany; 4 Department I for Internal Medicine, Faculty of Medicine and University Hospital Cologne, University of Cologne, Cologne, Germany; 5 Fraunhofer Institute for Translational Medicine and Pharmacology, Frankfurt, Germany; 6 German Centre for Infection Research (DZIF), partner site Bonn-Cologne, Cologne, Germany

**Keywords:** Bacteriophage, Phage therapy, Urinary tract infection, rUTI, Phage antibiotic synergy, Phage stability, Phage activity in urine, Biofilm degradation

## Abstract

Urinary tract infections (UTIs) are among the most common bacterial infections worldwide and are associated with high recurrence rates and substantial morbidity. New therapeutic approaches are urgently needed, especially for recurrent UTIs (rUTIs), among which phage therapy represents a promising option. Here, we systematically evaluated the performance of six *E. coli* phages under urinary tract–relevant conditions and established a quantitative area-under-the-curve (AUC)-based framework for the rational selection of therapeutic phages. Phage lysis behavior was tested under oxic and anoxic conditions, revealing modulation by oxygen availability. Phages were also screened for biofilm-degrading activity, and in silico depolymerase predictions were compared with in vitro results, showing strong correlation. Phage-antibiotic interactions and refined treatment strategies were further investigated. Phages G9062, G10400, and MM02 emerged as the most promising candidates, remaining stable and active in urine under physiological pH fluctuations and exhibiting synergistic effects with gentamicin and fosfomycin, but not with the bacteriostatic nitrofurantoin. Importantly, we demonstrate that treatment sequence is a critical determinant of efficacy, with phage administration prior to antibiotic exposure consistently producing the strongest synergistic effects. These findings highlight the potential of phages as an integral component of a holistic therapeutic strategy for rUTIs.

**Importance** Recurrent urinary tract infections (rUTIs) are a major clinical challenge characterized by repeated episodes, increasing antimicrobial resistance, and a substantial socioeconomic burden. Although bacteriophages represent a promising alternative or adjunct to antibiotics, their clinical translation has been hindered by the lack of standardized criteria for phage selection, dosing, and combination with antibiotics. This study addresses these gaps by defining key determinants of phage efficacy under conditions relevant to the urinary tract. Our findings highlight that phage performance is strongly shaped by the treatment environment and by interactions with antibiotics, and that inappropriate combinations or treatment sequences may compromise efficacy. By providing quantitative and transferable frameworks for evaluating phage effectivity, this work advances the rational development of phage-based therapies and helps bridge the gap between experimental phage research and clinically actionable treatment strategies for rUTI.

**Supplementary Information:**

The online version contains supplementary material available at 10.1186/s12866-026-05524-4.

## Highlights

Identification of *Escherichia coli* phages with therapeutic potential for recurrent urinary tract infections.

Phages exhibit stability and activity under conditions mimicking the urinary tract.

Urinary alkalinization does not appear to be required for phage activity.

Oxygen limitation modulates phage lysis dynamics.

Synergistic antibiotics (e.g., fosfomycin and gentamicin) potentiate phage-mediated bacterial clearance, whereas bacteriostatic agents (e.g., nitrofurantoin) attenuate phage efficacy.

Phage-first treatment sequencing maximizes phage–antibiotic synergy.

Novel quantitative AUC-based framework allows standardized comparison of phage lysis behavior across varying conditions.

Considerations for a multimodal management of recurrent urinary tract infections.

**Supplementary Information:**

The online version contains supplementary material available at 10.1186/s12866-026-05524-4.

## Introduction

Urinary tract infections (UTIs) are the second most common bacterial infection worldwide, affecting nearly 500 million people annually [1]. Recurrent UTIs (rUTIs) are a major global health problem, particularly among women, with 20–30% experiencing recurrence within 3–4 months of an initial episode [2]. They substantially reduce quality of life and impose an unsustainable economic burden on healthcare systems through repeated care, diagnostic testing, antibiotic use, and hospitalizations, with additional indirect costs from productivity loss. Together, these factors result in annual expenditures of billions of Euros, underscoring the urgent need for effective, non-antibiotic curative strategies [3]. The most frequently encountered pathogen responsible for both complicated and uncomplicated UTIs is *Escherichia coli (E. coli)* with the gastrointestinal tract serving as its major reservoir [3]. State-of-the-art therapy for cystitis caused by *E. coli* often involves antibiotics like fosfomycin or nitrofurantoin. Adjunctive measures include adequate fluid resuscitation, anti-inflammatory agents, behavioural interventions, and phytotherapeutics. While often effective in alleviating acute episodes, repeated courses of antibiotic agents or long-term antibiotic prophylaxis are a primary driver for antimicrobial resistance [4, 5]. Particularly in patients with rUTI, selection of resistant strains through repetitive antibiotic exposure and biofilm formation complicate effective treatment. These challenges underscore the urgent need for alternative therapeutic strategies for both treatment and prevention of rUTIs [6]. Bacteriophages (phages) are a promising option due to their self-amplification at the site of infection, host specificity, and ability to target antibiotic-resistant bacteria [7, 8]. They may also disrupt biofilms and are enriched in the mucosal surfaces [9], partly protecting them from washout during voiding [10]. Lytic phages have thus been explored as treatment supplements in UTI, applied mainly via oral or intravesical routes [11–14]. However, phage treatments have yielded mixed results, and the factors governing successful versus unsuccessful outcomes remain largely unresolved. This study systematically evaluated the in vitro therapeutic potential of bacteriophages for the management of urinary tract infections. By assessing host range, stability and activity in urine, interactions with antibiotics, and biofilm-degrading capacity, we identified phage candidates and refined treatment strategies in a preclinical setting that may serve as a basis for future clinical investigation.

## Results

### Identification of phages targeting uropathogenic E. coli (UPEC)

A previously identified and characterised cocktail of six phages selected from 43 candidates from the Leibniz Institue DSMZ (German Collection of Microorganisms and Cell Cultures GmbH) demonstrated favourable activity against extended-spectrum beta-lactamase (ESBL)-producing *E. coli* across a panel of 150 clinical isolates from diverse sources [15]. To determine which of these phages are most suitable for treating rUTIs, we assessed their host range against 25 clinically relevant urinary *E. coli* isolates. Phage activity on solid medium was quantified using efficiency of plating (EOP), defined as the ratio of the phage titer on a given test isolate relative to the titer on the propagation host [16]. The six phages displayed heterogeneous but overall broad host ranges, infecting between 24% and 48% of the UPEC panel. Four isolates could not be lysed by any of the tested phages. EOP distributions differed markedly between phages, with median values ranging from 0.06 for phage AC-L3 to 2.55 for phage G2494. Notably, phage G9062 exhibited significantly higher EOP values (mean = 1.5) than AC-L3 (mean = 0.06) and MM02 (mean = 0.08) (*p* = 0.02). Overall, phage G9062 demonstrated the broadest host range (48%) combined with a high median EOP of 1.5. In contrast, phages WFH and G2494 showed the narrowest host ranges (Supplementary Fig. 1).

### Phage stability testing under bladder-like conditions

**Fig. 1:**
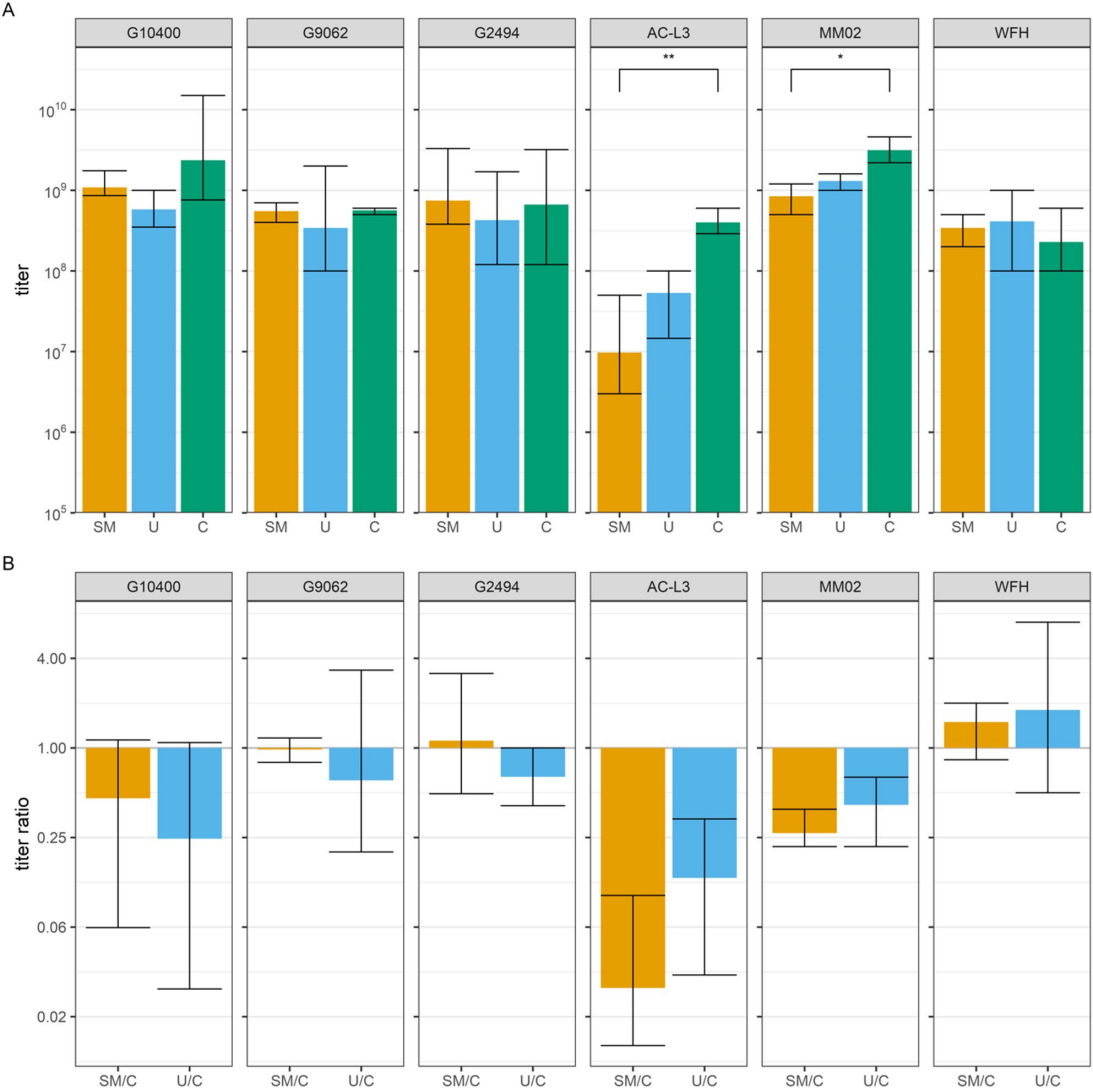
Stability of bacteriophages under conditions mimicking the human bladder. **A** Phage titers, expressed as plaque forming units (PFU/mL) were determined after 24 h incubation at 37 °C in SM buffer (SM) and in human urine (U). Phages stored at 4 °C in SM buffer served as controls (C). For AC-L3 and MM02, a significant reduction in titer was observed after incubation at 37 °C in SM buffer for 24 h. **B** Ratios A/C and B/C represent the titers after 24 h at 37 °C in SM buffer or urine, respectively, relative to the corresponding control titers at 4 °C in SM buffer. The Kruskal-Wallis test, followed by Dunn’s test yielded *P* values of < 0.05 (*) and < 0.01(**)

To evaluate whether the selected phages are suitable for intravesical therapy, their stability was assessed under conditions mimicking the human bladder. Each phage was incubated for 24 h at 37 °C either in sodium chloride–magnesium sulfate (SM) buffer or in pooled female urine, and compared to a control group stored at 4 °C in SM buffer. Following incubation, phage titers were determined on their respective host strains, and stability was expressed as the ratio of titers after incubation to those of the corresponding stored controls. Titer reductions were observed across conditions only for phages AC-L3 and MM02; no significant differences were detected for the other phages tested. Specifically, incubation at 37 °C in SM buffer resulted in an approximately 1-log decrease in titers for both phages compared with the control condition. These differences were statistically significant (AC-L3: *p* < 0.02; MM02: *p* = 0.01). Incubation at 37 °C in urine was associated with less pronounced impairment of phage stability than incubation in SM buffer at the same temperature. For the remaining phages, no significant differences in titers were observed across conditions, indicating that most phages maintain stability during 24 h of exposure to urine at body temperature (Fig. 1).

### Phage activity in urine

**Fig. 2:**
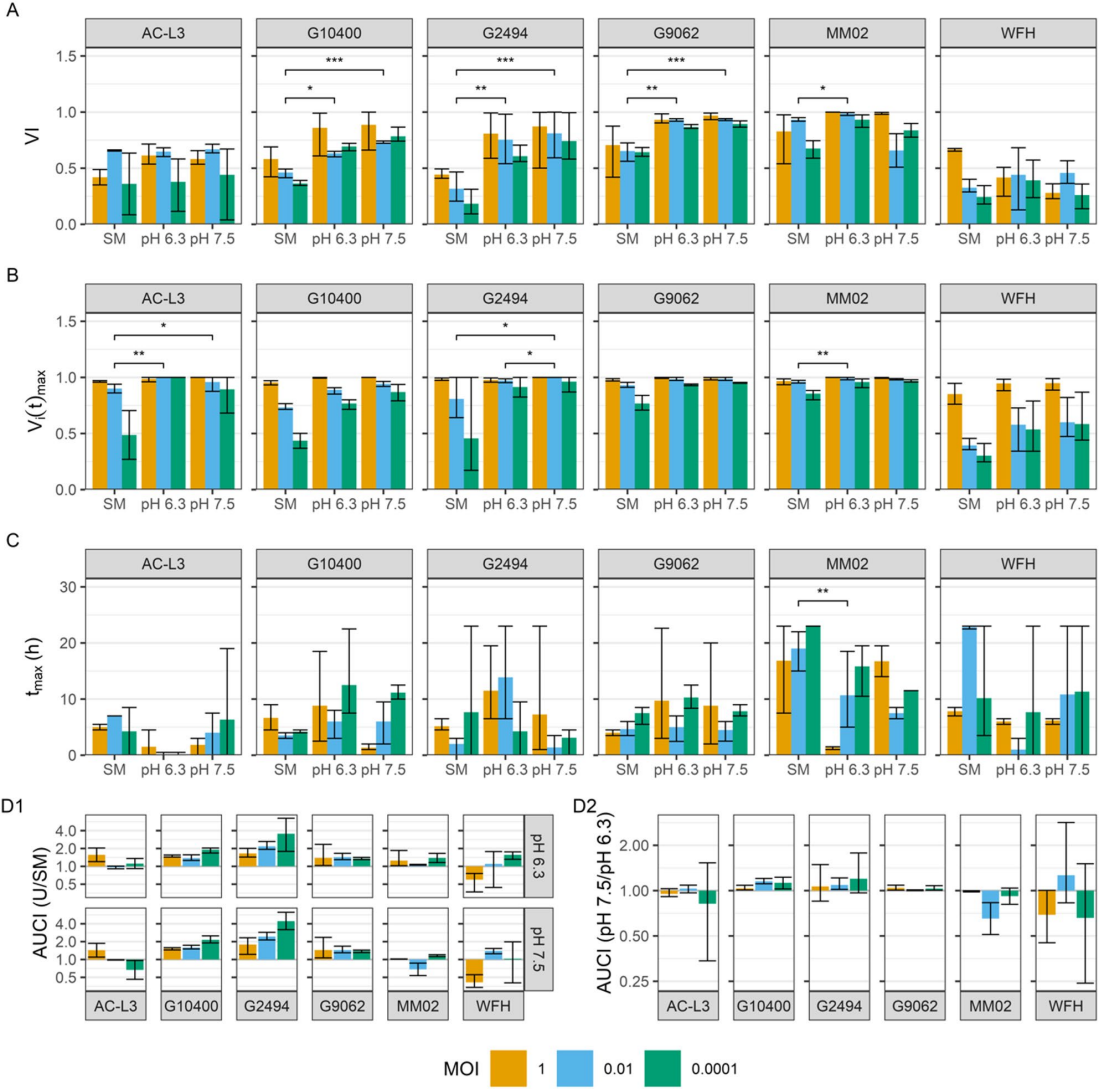
Phage lysis behaviour in SM buffer and urine. Calculations are based on bacterial reduction curves for phages AC-L3, G10400, G2494, G9062, MM02, and WFH with their respective propagation hosts in SM buffer and urine under native conditions (pH 6.3) and after artificial pH adjustment to pH 7.5. **A** Virulence index (VI) determined in SM buffer (VI_SM_ = 1 – A_SM_,_10_^x^/A0_SM_), urine pH 6.3 (VI_U6.3_ = 1 – A_U6.3,10_^x^/A0_U6.3_), or urine pH 7.5 (VI_U7,5_ = 1 – A_U7.5,10_^x^/A0_U7.5_), (**B**) Maximum of temporal virulence (Vi(t)_max_), and (**C**) timepoint of maximum temporal virulence (t_max_). **D.1**AUC indices (AUCI) comparing phage activity in urine to activity in SM buffer at a defined MOI (AUCI_(U6.3/SM),10_^x^ = VI_U6.3,10_^x^/VI_SM,10_^x^ or AUCI_(U7.5/SM),10_^x^ = VI_U7.5,10_^x^/VI_SM,10_^x^). **D.2** AUCI assessing the effect of urine alkalinisation (AUCI_(U7.5/U6.3),10_^x^ = VI_U7.5,10_^x^/VI_U6.3_^x^). Asterisks denote statistical significance calculated across all three MOIs. The Kruskal-Wallis test, followed by Dunn’s test yielded *P* values of < 0.05 (*), < 0.01(**), and < 0.001 (***). Abbreviations: PC, positive control; VI, virulence index; Vi(t)_max_, maximum of temporal virulence; t_max_, timepoint of maximum temporal virulence; AUCI, AUC index; SM, SM buffer; U, urine; A, area under the curve; A0, AUC of positive control; 10^x^, used MOI; U_6.3_, urine at pH 6.3; U_7.5_, urine at pH 7.5

Building on the stability assessment described above, we evaluated phage lytic activity in human urine under physiologically relevant conditions. Stability was defined as the ability of phages to maintain viability under perturbing conditions and to recover without loss of function, whereas lytic activity refers to the capacity to lyse target bacteria under the respective conditions. Based on previous reports indicating that urine may impair phage activity compared with standard phage buffer [17, 18], lytic activity was quantified at three multiplicities of infection (MOI 1, 0.01, 0.0001), representing high to low phage-to-host ratios, in pooled female urine (pH 6.3) and in SM buffer. Lytic activity was quantified based on parameters derived from the area under the curve (AUC) of bacterial growth curves (Methods: Virulence and AUC indices). Whereas the virulence index (VI) was used to assess phage virulence at a defined MOI in either SM buffer or urine, the AUC index (AUCI) enabled direct comparison of phage activity across both conditions at the same MOI. AUCI values < 0.75 were considered indicative of reduced urinary activity, whereas values > 1.25 indicated enhanced activity. Temporal virulence metrics, including the time-dependent virulence index (VI(t)) and the timepoint of maximal virulence (t_max_), enabled assessment of the kinetics of phage-mediated lysis, capturing the progression of lytic activity over time. Overall, phage MM02 showed the strongest lytic activity in urine, with the highest absolute virulence index (VI = 1 at an MOI of 1) and maximum temporal virulence (Vi(t)_max_ = 1 at an MOI of 1). Notably, virulence peaked substantially earlier in urine than in SM buffer, with t_max_ occurring at 1.33 h compared with 16.3 h (Fig. 2A-C). While phage MM02 exhibited the strongest absolute lytic effect in urine, the activity of phage G2494 was most positively influenced by urine, represented by the highest AUCI values (AUCI = 0.75 at an MOI of 1) (Fig. 2D1). Except for phage WFH at a MOI of 1, all phages displayed neutral or enhanced lytic activity in urine (pH 6.3) relative to SM buffer, particularly at low MOIs, as visualized by the AUCI. The effect was statistically significant for phages G10400, G2494, G9062, and MM02 (*p* = 0.047). Furthermore, especially at higher MOIs, bacterial regrowth could be delayed in urine for all phages except AC-L3 and WFH (Supplementary Fig. 2). Of note, independent of the growth condition, the highest MOI did not necessarily yield the best overall growth impairment. Although an MOI of 1 initially produced efficient lysis, as reflected by high Vi(t)_max_ values and an early t_max_, bacterial regrowth occasionally occurred earlier than at lower MOIs and, in some cases, led to higher endpoint optical densities, resulting in a lower VI over 24 h.

To evaluate whether urine alkalinisation enhances phage activity, we performed an additional experiment in which pooled urine (natural pH 6.3) was adjusted to pH 7.5 (as used in phage buffer) with potassium hydroxide. Determination of the AUCI_(U7.5/U6.3),10_^x^ revealed a modest increase in activity after pH increment across all tested MOIs for phages G10400 (mean over all MOI’s 1.10), G2494 (1.10), and G9062 (1.03), reaching statistical significance for G10400 and G9062 (*p* = 0.02). In contrast, phages AC-L3, MM02, and WFH showed heterogeneous responses, with an overall non-significant decrease in activity after urine alkalinization (Fig. 2 C2).

### Phage activity under anoxic conditions

We explored the effect of an anoxic environment on the activity of phages G9062, which exhibited the largest host range on UPEC in previous experiments, and phage MM02, which showed the best lytic behaviour in urine. Infection curves were conducted under oxic and anoxic conditions with diverse UPEC strains: UPEC 0310 being susceptible for both phages G9062 and MM02, UPEC 8457 showing susceptibility towards G9062 only, and UPEC 3603 that generated no plaques with the double agar overlay (DAO) method neither for G9062 nor for MM02. A laboratory *E. coli* strain (W3110), included for comparison, showed resistance to phage G9062 and susceptibility to MM02. A comparative analysis was performed based on the virulence index and temporal virulences (Methods: Virulence and AUC indices). Negative virulence indices may arise in phage–host combinations that are non-susceptible and should be interpreted accordingly. Because calculating AUC indices from negative virulence values requires a different approach, these data were not included in the visualizations presented here. When phages are avirulent towards a bacterial strain, bacterial growth comparable to or slightly exceeding the growth control may be observed. Possible explanations include degradation of phage particles, providing additional nutrients, or phage binding without lysis leading to altered bacterial metabolism.

**Fig. 3:**
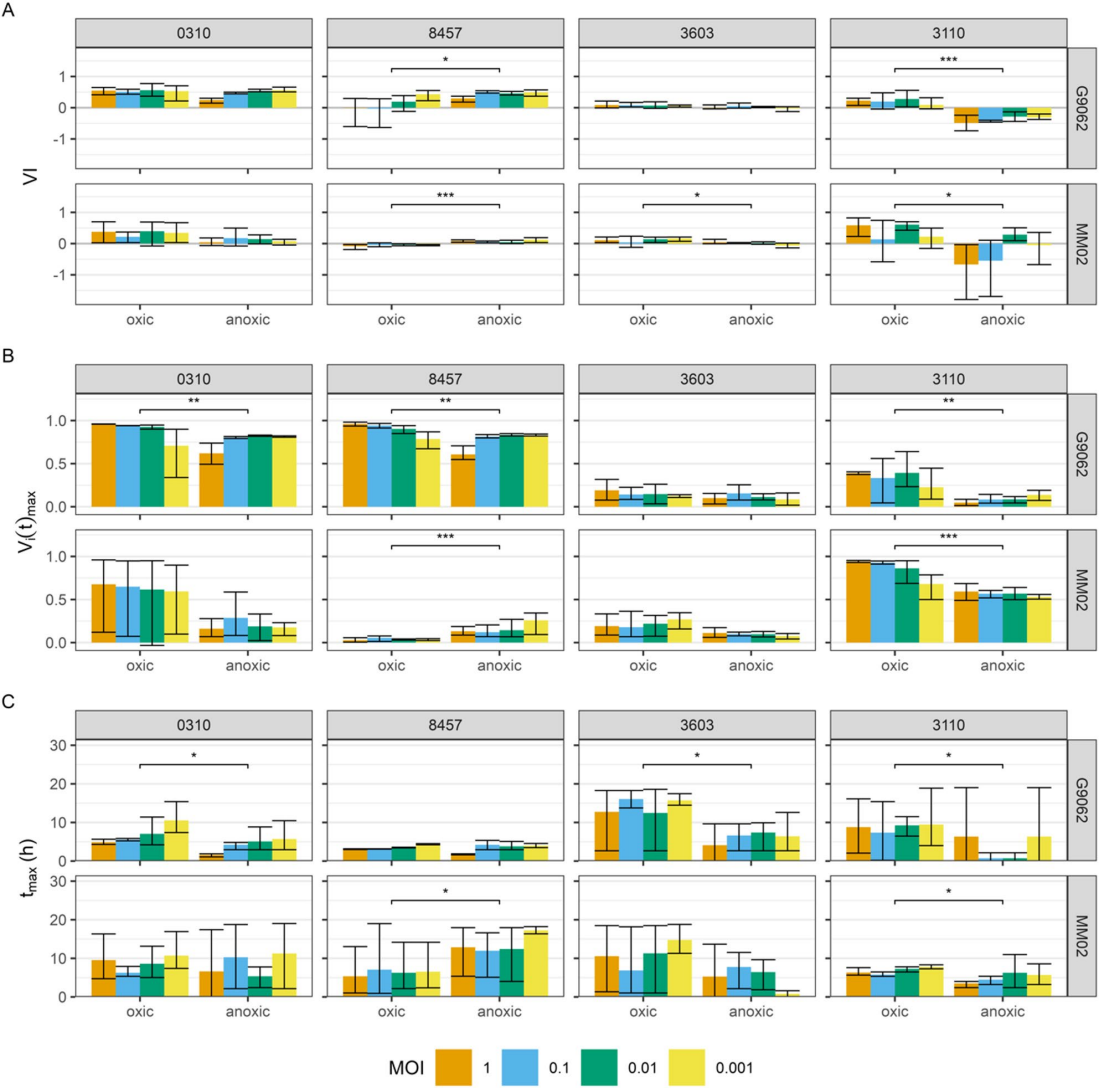
Comparative overview of phage virulence under oxic and anoxic conditions. Calculations are based on bacterial reduction curves for phages G9062 and MM02 with UPEC 0310, UPEC 8457, UPEC 3603 and *E. coli* W3110 under oxic or anoxic growth conditions. **A** Virulence index (VI_ox_ = 1 – A_ox,10_^x^/A0_ox_; VI_anox_ = 1 – A_anox,10_^x^/A0_anox_), (**B**) maximum of temporal virulence (Vi(t)_max_), and (**C**) timepoint of maximum temporal virulence (t_max_) for phages G9062 and MM02 under varying oxygen environments. Asterisks denote statistical significance calculated across all three MOIs. Asterisks denote statistical significance calculated across all MOIs. The Mann-Whitney-Wilcoxon test yielded P values of < 0.05 (*), < 0.01(**), and < 0.001 (***). Abbreviations: PC, positive control; VI, virulence index; Vi(t)_max_, maximum of temporal virulence; t_max_, timepoint of maximum temporal virulence

In general, the lysis behavior of the phages G9062 and MM02 was altered under anaerobiosis compared to oxic growth conditions in a host-strain-dependent manner (Fig. 3; Supplementary Fig. 3). While phage G9062 showed a robust virulence index with UPEC 0310 under both conditions and over the whole range of MOIs, the virulence index with UPEC 8457 improved even slightly under oxygen-deprived conditions, reflecting increased host susceptibility. Notably, the Vi(t)_max_ values were reached earlier (indicated by lower t_max_), but were slightly lower under anaerobiosis. For non-susceptible UPEC 3603 and *E. coli* W3110, the already low virulence indices decreased further under anoxic conditions, reaching statistical significance for W3110 (*p* < 0.001). However, Vi(t)_max_ of G9062 towards UPEC 3603 showed a slight improvement. Nevertheless, the overall low virulence of the phage towards the two non-susceptible hosts remained low, which is underlined by the large distribution of t_max_ values at different MOIs.

In comparison, the phage virulence of MM02 was significantly affected by anoxic growth conditions towards all *E. coli* isolates tested. For most hosts, Vi(t)_max_ decreased significantly under anoxia. However, sensitivity towards UPEC 8457 increased, shifting from non-infective to virulent, as further supported by a significantly elevated virulence index (*p* < 0.001). Interestingly, although MM02 was less virulent towards both UPEC 0310 and *E. coli* W3110, the significant shift in t_max_ for both strains trended contrarily: Vi(t)_max_ under anoxic conditions was reached later with UPEC 0310, but earlier with *E. coli* W3110.

Taken together, these findings indicate that anoxic cultivation distinctly shaped the lysis profiles of the investigated phages, modulating their activity in a strain-dependent manner. While G9062 retained robust performance under both oxygen regimes, MM02 responded strongly to anaerobic host metabolism, making it a less reliable candidate for targeting UPEC under fluctuating environmental conditions.

### Correlation of in silico prediction of depolymerase enzymes with in vitro testing of biofilm degradation

**Fig. 4:**
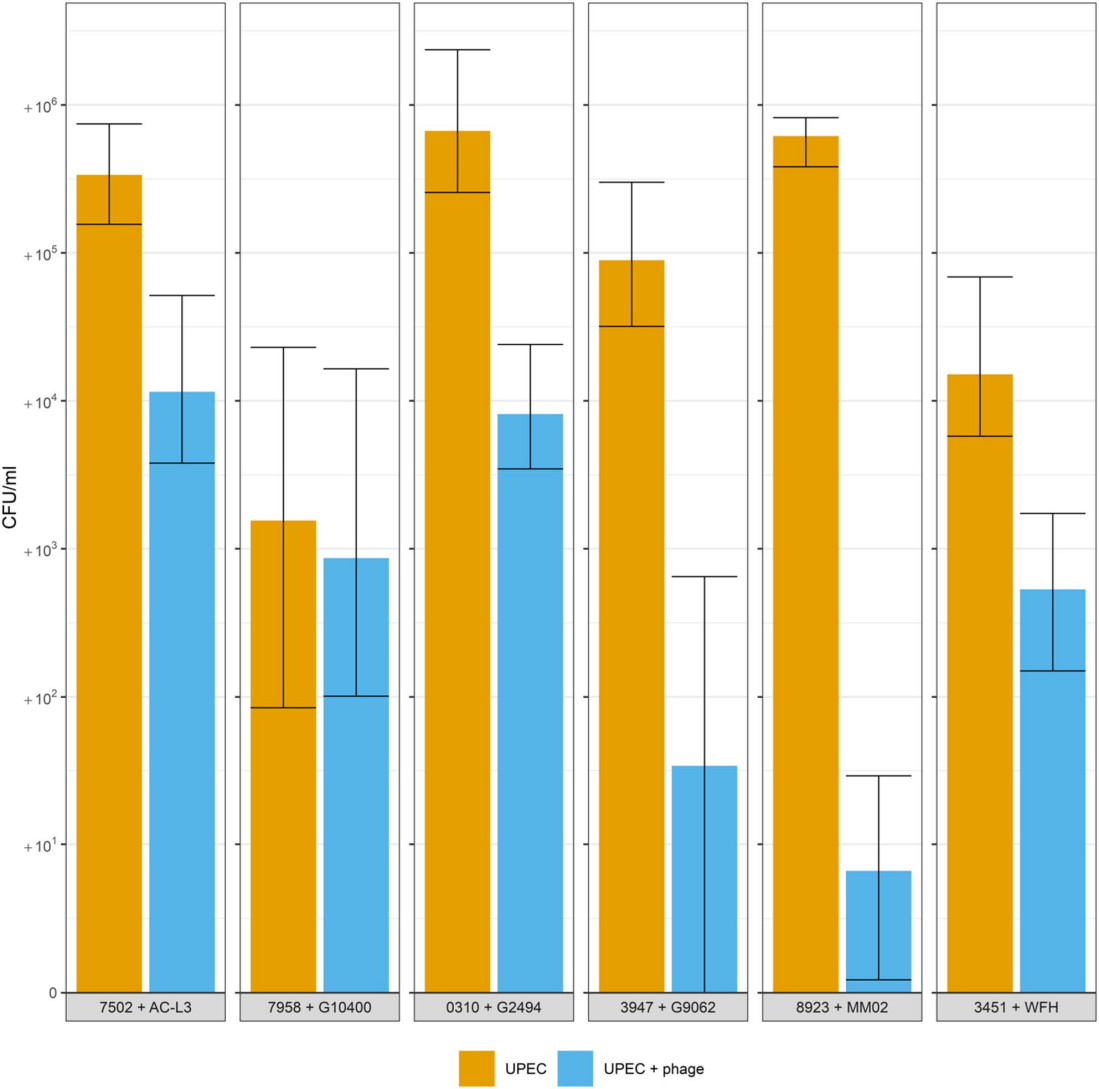
Phage in vitro biofilm reduction. Mature phage susceptible UPEC biofilms grown over 48 h were exposed to matching phages at an MOI of 10 for 12 h. Viable biofilm cell numbers are indicated in CFU/mL compared to untreated positive controls

Indwelling urinary catheters frequently promote biofilm development, leading to infections that are challenging to manage and usually necessitate device removal. Some phages express depolymerases that facilitate the disruption of established biofilms. The six *E. coli* phage genomes were screened for depolymerase-encoding genes using two machine-learning prediction tools (PhageDPO [19] and DePP [20]), supplemented by manual annotation. Outputs from both algorithms were largely concordant, whereas targeted keyword-based inspection enabled the detection of additional putative depolymerase genes. In total, phages G9062 and MM02 exhibited the greatest genotypic potential for biofilm degradation, carrying 9 and 10 predicted depolymerases, respectively (Supplementary Table 1). To evaluate whether these in silico predictions translate into phenotypic activity, the biofilm-disrupting potential of the six *E. coli* phages was assessed against their corresponding phage-susceptible UPEC isolates using minimum biofilm eradication concentration (MBEC) plates. Human urine was used for the biofilm assays to better mimic the physiological conditions of urinary biofilms, that are strongly influenced by environmental factors such as nutrient availability, pH, and host-derived components. The MBEC assay does not directly assess degradation or removal of the biofilm matrix but quantifies the number of viable bacteria that remain associated with the biofilm following treatment. Residual biofilms were mechanically disrupted by sonication after phage exposure, and surviving cells were enumerated by counting colony forming units (CFU). All phage-treated MBEC pegs exhibited reduced CFU counts relative to untreated controls, although the magnitude of the effect varied substantially among phages. Consistent with the in silico predictions of depolymerase-encoding genes, MM02 achieved the strongest reduction, lowering viable biofilm-associated cells by more than 4 log CFU/mL, closely followed by G9062 with approximately a 3 log CFU/mL decrease (Fig. 4). In contrast, phage G10400 showed limited activity in the MBEC assay, indicating that its biofilm-associated killing capacity was overestimated based on genomic predictions alone. In silico analyses predict the presence of putative depolymerase genes but do not account for their expression, secretion, or enzymatic activity. To further assess depolymerase expression at the phenotypic level, halo formation on bacterial lawns was used as an indirect proxy. Halo presence and diameter were quantified on DAO plates using the same UPEC isolates as in the MBEC assays. Phages MM02 and G9062 produced the largest and most distinct halos (2.5 and 2 mm, respectively), supporting active depolymerase expression and aligning with their superior performance in reducing viable biofilm-associated cells (Supplementary Table 2).

### Phage antibiotic interactions (PAI)

**Fig. 5:**
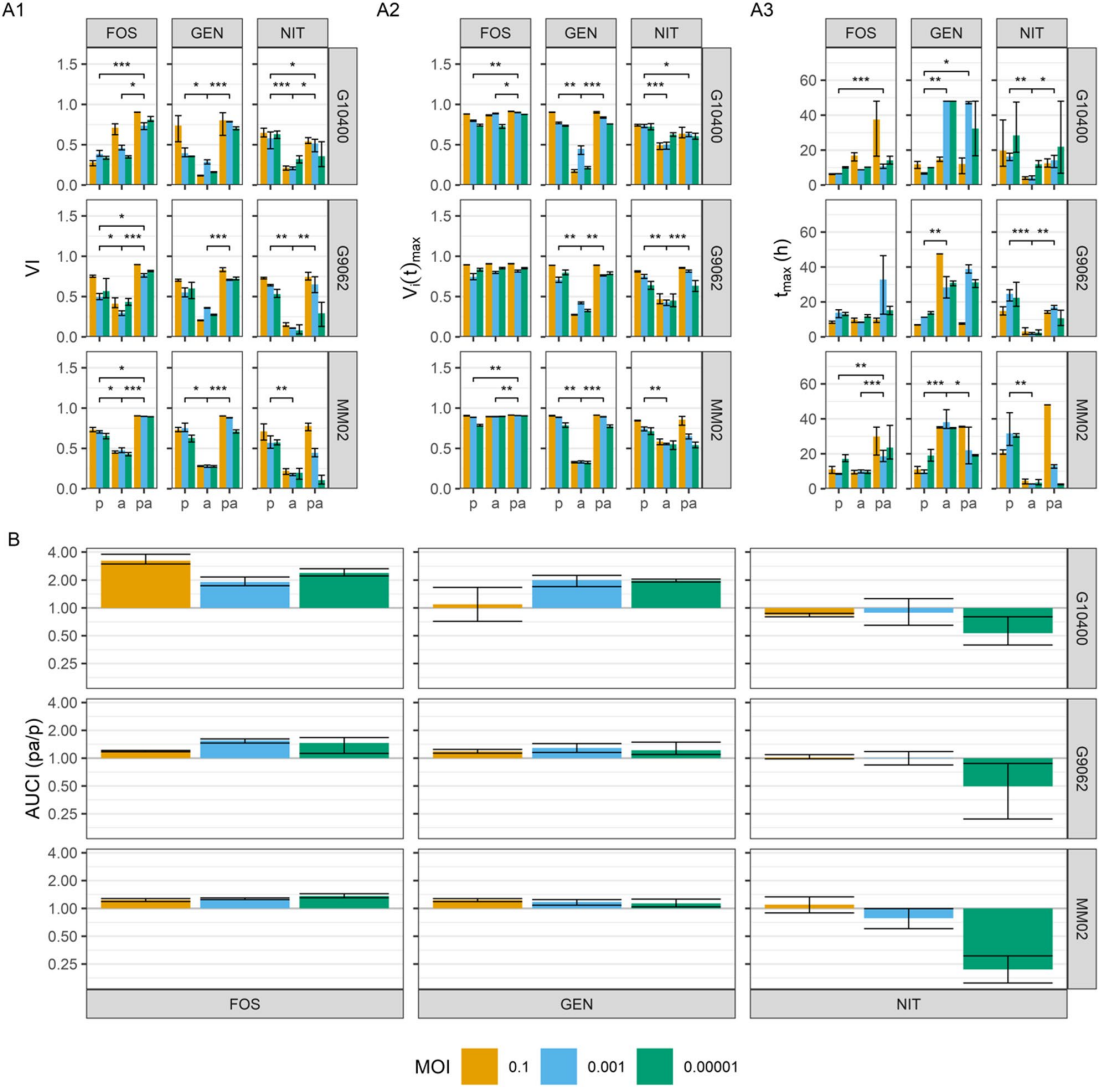
Phage antibiotic interactions. Calculations are based on bacterial reduction curves for fosfomycin, gentamicin or nitrofurantoin at 0.25 MIC with phages G9062, G10400 or MM02 on their propagation hosts at MOIs ranging from 0.1, 0.001 to 0.00001 (**A1**) Virulence index (VI) assessing killing potential for either antibiotic (a) alone at 0.25 MIC, phage (p) alone at a given MOI (10^x^) or the combination of both (VI_a_ = 1 – A_a,10_^x^/A0; VI_p_ = 1 – A_p,10_^x^/A0 ; VI_p.a._ = 1 – A_p.a.,10_^x^/A0), (**A2**) maximum of temporal virulence (Vi(t)_max_), and (**A3**) timepoint of maximum temporal virulence (t_max_). **B** AUC index (AUCI) comparing phage antibiotic combinations to phage only (AUCI_(pa/p),10_^x^ = VI_p.a.,10_^x^/VI_p,10_^x^). Asterisks denote statistical significance calculated across all three MOIs. The Kruskal-Wallis test, followed by Dunn’s test yielded *P* values of < 0.05 (*), < 0.01(**), and < 0.001 (***). Abbreviations: PC, positive control; FOS, fosfomycin; GEN, gentamicin; NIT, nitrofurantoin; VI, virulence index; Vi(t)_max_, maximum of temporal virulence; t_max_, timepoint of maximum temporal virulence; AUCI, AUC index; a, antibiotic; p, phage; p.a., phage antibiotic combination

A major limitation of phage therapy is the emergence of phage-resistant bacterial mutants, which can be mitigated through combined treatment with antibiotics [21]. We evaluated efficiency of the three most promising *E. coli* phages identified in earlier assays (G9062, G10400, and MM02) in combination with three antibiotics commonly used to treat UTIs. Prior to assessing phage-antibiotic interactions, minimum inhibitory concentrations (MICs) of gentamicin, fosfomycin, and nitrofurantoin were determined for each host strain (Supplementary Table 3). Phage antibiotic interactions were analyzed with the planktonic phage activity assay (PKA) using phages G9062, MM02 and G10400 at MOIs ranging from 0.1, 0.001 to 0.00001 and antibiotics at 0.25 MIC. LB medium served as the growth medium to ensure standardized and reproducible growth conditions and to minimize environmental variability. As antibiotic activity and bacterial growth kinetics can vary in urine due to donor-dependent differences in composition, the use of LB allows reliable interpretation of synergy or antagonism effects by ensuring that observed outcomes are less influenced by fluctuations in medium composition and more reflective of phage–antibiotic effects under standardized conditions. Bacterial growth inhibition was monitored via turbidity measurements, allowing assessment of the combined effects of phages and antibiotics at sub-inhibitory concentrations. Quantification of the PKA results was based on metrics derived from the area under the bacterial growth curve over 48 h, as described in the methods section. Comparing the virulence indices of phage-antibiotic combinations to those of phage-only treatments at defined MOIs generated the AUCI, which reflects the relative effectiveness of the combination. As no broadly accepted AUCI thresholds for classifying phage–antibiotic interactions currently exist, cut-off values were defined empirically based on the distribution of the experimental data and the biological interpretation of the observed treatment effects. AUCI values > 1.25 were classified as synergistic, values between 0.75 and 1.25 as neutral, and values < 0.75 as antagonistic. Analysis revealed neutral or synergistic interactions between all tested phages with both fosfomycin and gentamicin. Of note, although combinations with these antibiotics were favorable, as indicated by increased VI and AUCI values compared to phage only treatments, maximal bacterial growth inhibition was reached at later time points, as reflected by an elevated t_max_, while Vi_(tmax)_ remained largely unaffected (Fig. 5). In addition to reducing optical density, phage-antibiotic combinations substantially delayed bacterial regrowth. For example, for G9062 at an MOI of 0.001 on its host DSM 103,465, regrowth was delayed from 15 h with phage alone to 35 h when combined with fosfomycin at 0.25× MIC. In several cases, no regrowth was observed for the duration of the experiment in the combination treatments (Supplementary Fig. 4).

In contrast to gentamicin and fosfomycin, combining phages with nitrofurantoin proved disadvantageous. Interestingly, PAI were dose dependent. If applied at a high MOI of 0.1, the combination of nitrofurantoin and phage was neutral (phages G9062 and MM02) or antagonistic (phage G10400). With decreasing MOIs (from 0.001 to 0.00001), a combination consistently resulted in antagonistic effects (Fig. 5).

**Fig. 6:**
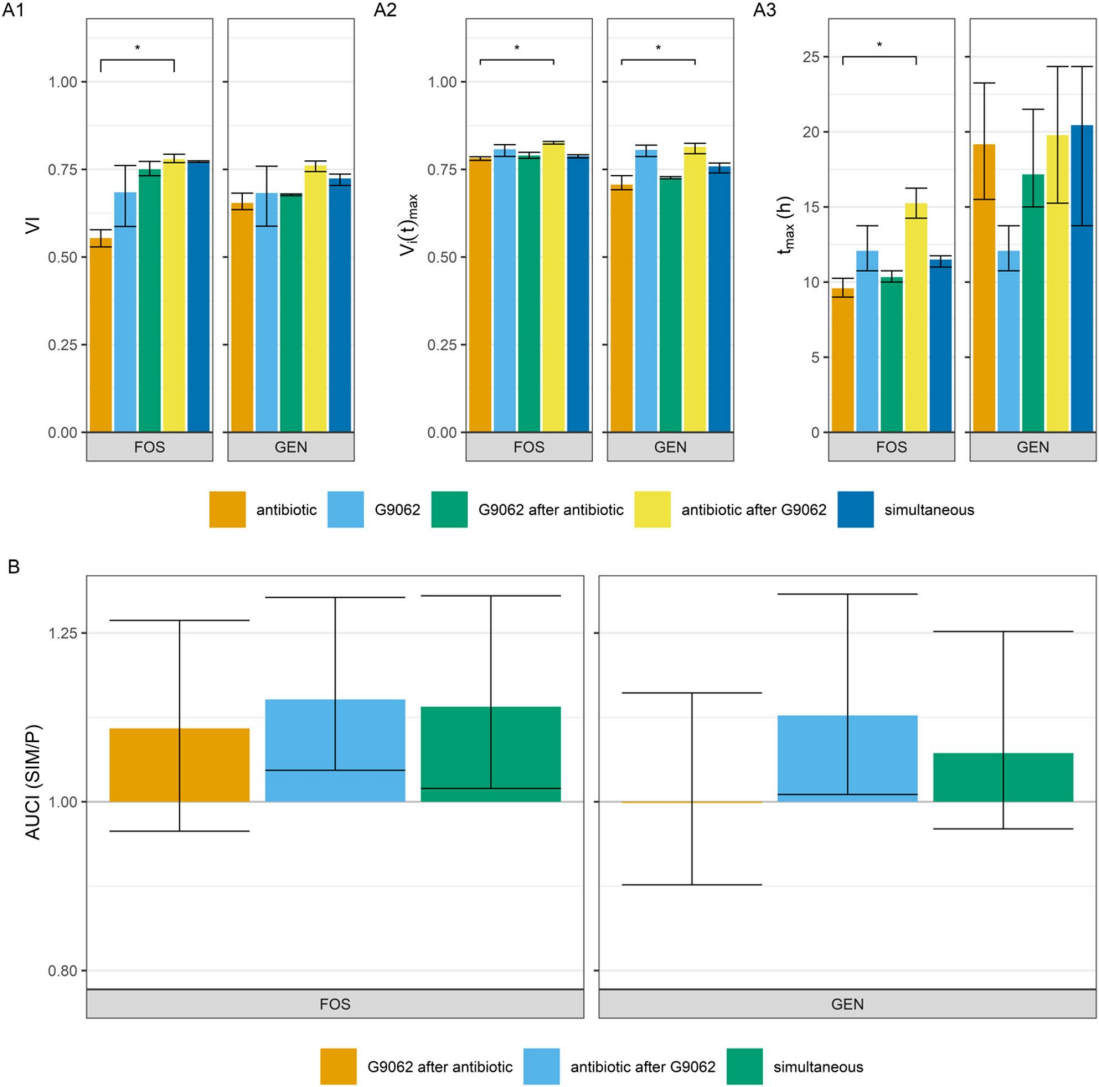
Phage antibiotic treatment sequence. Calculations are based on bacterial reduction curves for different treatment schedules of fosfomycin or gentamicin at 0.25 x MIC with phage G9062 on its propagation host DSM 103,265: simultaneous combination (SIM), phage addition 4 h before antibiotics (PbA), and phage addition 4 h after antibiotics (PaA). **A1** Virulence index (VI) assessing killing potential for the SIM, PbA or PaA regimens at an MOI of 0.001 (VI_SIM_ = 1 – A_SIM,10_^3^/A0; VI_PbA_ = 1 – A_PbA,10_^3^/A0 ; VI_PaA_ = 1 – A_PaA,10_^3^/A0). Shown are also (**A2**) the maximum of temporal virulence (Vi(t)_max_), and (**A3**) timepoint of maximum temporal virulence (t_max_). **B** AUC index (AUCI) comparing combined treatment sequences to phage (p) only (AUCI_(SIM/p),10_^3^ = VI_SIM,10_^3^/VI_p,10_^3^; AUCI_(PbA/p),10_^3^ = VI_PbA,10_^3^/VI_p,10_^3^; AUCI_(PaA/p),10_^3^ = VI_PaA,10_^3^/VI_p,10_^3^). Asterisks denote statistical significance calculated across all three MOIs. The Kruskal-Wallis test, followed by Dunn’s test yielded P values of < 0.05 (*). Abbreviations: PC, positive control; FOS, fosfomycin; GEN, gentamicin; NIT, nitrofurantoin; VI, virulence index; Vi(t)_max_, maximum of temporal virulence; t_max_, timepoint of maximum temporal virulence; AUCI, AUC index; p, phage; SIM, simultaneous treatment

Subsequently, we investigated the optimal timing for antibiotic addition in combinations previously shown to act synergistically with phage G9062. Liquid cultures were exposed to phage application before antibiotic addition (PbA), simultaneous application (SIM), and phage addition after antibiotic treatment (PaA). Resulting growth curves were compared with untreated controls as well as phage-only and antibiotic-only conditions. To quantify treatment efficacy, virulence and AUC indices were calculated for the period between 4 and 24 h. The PbA regimen—phage applied 4 h before antibiotics—produced the strongest reduction in bacterial growth, with the SIM condition yielding the next-best outcome (Fig. 6). These findings were corroborated by bacterial enumeration after 24 h of incubation in the Tecan Infinite Microplate Reader. Endpoint survival correlated with the decline in turbidity and was lowest in the PbA group compared with SIM or PaA treatments (Supplementary Fig. 6).

## Discussion

Urinary tract infections (UTIs) are among the most common bacterial infections, particularly in women. Their frequent recurrence presents a therapeutic challenge, as it places a substantial burden on patients and repeated antibiotic exposure contributes to the development of antimicrobial resistance (AMR). In this context, bacteriophages represent a promising alternative or adjunctive therapeutic strategy, as antibiotic-resistant bacteria often remain susceptible to phage-mediated lysis with high strain specificity.

In this study, we identified bacteriophages with potential therapeutic utility against uropathogenic *Escherichia coli* by systematically evaluating host range, stability and activity under physiologically relevant conditions, and biofilm-degradation capacity. In addition, we explored combinations of phages and antibiotics to assess their potential for future combination therapies.

Our analytical framework builds upon the Virulence Index proposed by Storms et al. [22] by extending AUC-based virulence quantification beyond single-condition measurements. Whereas the original Virulence Index was developed to quantify phage virulence under defined experimental conditions, the here proposed AUCI incorporates condition-specific analyses, enabling systematic comparison of phage performance across physiologically relevant environments and treatment regimens. Incorporation of temporal virulence profiles [VI(t)] captures lysis dynamics not resolved by endpoint AUC metrics, while retention of MOI-specific information preserves biologically relevant differences that may be obscured by global aggregation. Collectively, these extensions expand virulence assessment from a descriptive characterization tool to a framework for comparative, multi-condition evaluation of phage efficacy with direct relevance for therapeutic phage selection.

Placing our findings within the current pathomechanistic understanding of rUTI, and in light of evidence from two randomized controlled trials (RCTs) [23, 24], as well as multiple case reports and case series [8, 25], we propose a conceptional multimodal treatment framework for *E. coli*-induced rUTI. The approach outlined here is intended to integrate the findings of the present in vitro study with the existing clinical literature and should be regarded as hypothesis-generating rather than as a clinically validated treatment strategy. While our experimental models provide insight into phage behavior under physiologically relevant urine and bladder-like conditions, they do not fully recapitulate the complexity of rUTI in vivo.

A key prerequisite for successful phage therapy in rUTI is the culture-based detection of phage-susceptible *E. coli* from urine samples and the subsequent matching to suitable therapeutic phages. Among the phages investigated, G9062 displayed the broadest host range against UPEC isolates (48%) and showed high efficiency of plating, underscoring its potential clinical relevance. Noteworthy, host ranges differed between the UPEC panel used in this study and a previously analyzed collection of clinical *E. coli* isolates from various sources (primarily blood and stool). For example, phage G9062, which demonstrated a large host range against UPEC (48%), was less active against *E. coli* of diverse origins (27%) [15]. This discrepancy highlights the importance of carefully selecting representative bacterial panels to obtain accurate information specific to the targeted infectious disease.

Another cornerstone of successful phage therapy is the assurance of sufficient phage stability under human physiological conditions. All tested phages remained stable for at least 24 h under conditions simulating the bladder environment. Although two phages (AC-L3 and MM02) showed a ~ 1-log titer reduction at 37 °C in SM buffer, this loss was not observed when they were incubated in urine, indicating good stability in the actual treatment environment. Since intravesically administered phages are cleared within hours due to voiding and therefore require repeated dosing, this demonstrated urine stability supports their clinical utility for UTI treatment. For uncomplicated rUTIs, phage delivery via intermittent catheterization with single-use catheters has been used, with patients retaining the solution as long as possible before voiding [8, 22, 23, 25]. While optimal dosing regimens are not yet established, prior studies suggest that doses of 10⁹–10¹¹ PFU administered once or twice daily for 3–7 days are feasible and appear reasonable [23, 24].

Phages must not only remain stable but also retain their lytic activity in the urinary tract, therefore their planktonic lytic activity in pooled female urine was evaluated. Contrary to earlier concerns about reduced phage efficacy in urine [17, 18], most phages showed increased antibacterial activity in urine compared to standard SM buffer, with phage MM02 performing best, followed by G9062. The study also examined the effect of phage dosing and found that high multiplicities of infection (MOI 1) did not consistently produce sustained bacterial growth inhibition. Although higher MOIs led to strong initial bacterial reduction, early bacterial regrowth was sometimes observed in both urine and SM buffer. This regrowth appeared to depend more on the specific phage–host combination than on the growth environment, likely due to increased selection pressure at high MOIs that accelerates the emergence of phage-resistant mutants. These findings highlight the need for careful dose selection in phage therapy. While high phage titers can provide rapid initial killing, moderate titers may offer more durable bacterial control and avoid drawbacks associated with high doses, including reduced phage tolerance [24] and potentially stronger immune responses [26].

The median pH of 24-h urines is approximately 6, but it can fluctuate between 5.5 and 8.0 depending on time of day, diet, and infection status [27]. Consequently, phage activity must remain robust across a range of pH values. As early as 1936, Michon recommended urine alkalinisation before phage therapy for UTI [28] as the pH value was already known to impact phages. However, to our knowledge, no systematic investigation has been conducted to substantiate this recommendation. In this work, adjusting urine pH from 6.3 to 7.5 produced only modest, phage-dependent effects on lytic activity, with slight increases for G10400, G2494, and G9062, but heterogeneous and overall non-significant decreases for AC-L3, MM02, and WFH. Therefore, generalized urine alkalinization cannot be recommended for phage treatment of UTIs without prior in-vitro testing. These observations align with reports that most phages remain infectious within moderate pH ranges (approximately 5–9) and that pH sensitivity is phage-dependent [29, 30].

Since the gastrointestinal tract represents a major source and reservoir of *E. coli*, with intestinal colonization serving as a potential driver of reinfection [3], it has been suggested to add oral phages for secondary prevention if the target *E. coli* can be isolated from stool—e.g., 10⁹–10¹¹ PFU per dose twice daily for 5–7 days [31]. In contrast to intravesical instillation, oral phage preparations should be encapsulated or combined with an antacid to reduce phage inactivation in the acidic gastric environment, which is considerably more acidic than urine [30, 32]. Anaerobic growth conditions are encountered both in the gut, the major reservoir of UPEC, and in the urinary tract, where inflammation and bacterial overgrowth may produce locally anoxic microenvironments [33, 34]. Phages aimed at treating rUTI and enabling intestinal decolonization for secondary prevention must therefore remain effective in this context. Oxygen limitation induces metabolic and molecular changes in facultatively anaerobic bacteria such as *E. coli*, which can alter the expression and accessibility of phage receptors (e.g., OmpC, OmpF) and thereby affect infection dynamics [34–36]. Indeed, studies on *Salmonella* phages have shown that oxygen absence results in a longer latent period and smaller burst size, impeding lytic activity, but can also reduce the development of bacterial resistance [35, 37]. The findings of this study demonstrated that anoxia modulates phage lysis profiles in an isolate-dependent manner. While phage G9062 appeared resilient to oxygen limitation, phage MM02 exhibited pronounced oxygen dependence, suggesting limited reliability in fluctuating redox environments such as the gut or urinary tract. These results underscore the importance of incorporating physiologically relevant oxygen conditions into preclinical phage screening to ensure the effective selection of candidates for intestinal or intravesical therapy.

Especially for catheter-associated UTIs, the biofilm-degrading capacity of certain phages represents an important additional asset. Since the presence and activity of depolymerases can vary widely among phages, selecting the most effective biofilm-degrading candidates is crucial. However, traditional in vitro testing is time- and resource-intensive, making computational approaches increasingly valuable. We compared in silico depolymerase gene predictions [19, 20] with in vitro biofilm degradation and plaque halo size, as halo formation reflects enzymatic breakdown of extracellular polymeric substances [38, 39]. In silico predictions correlated strongly with plaque halo size and the actual reduction of viable UPEC biofilm cells. Among them, phage MM02 emerged as the most promising candidate, followed by G9062. Overall, these findings suggest that combining genetic depolymerase prediction with halo assessment provides an efficient preliminary screening strategy for identifying anti-biofilm phages and can guide more resource-intensive in vitro testing.

Bacteriophages offer a targeted strategy for combating antibiotic-resistant pathogens and can potentiate antibiotic activity through synergistic interactions [39–42]. Using planktonic phage activity assays, we examined the interactions between phages and the antibiotics gentamicin, fosfomycin, and nitrofurantoin. All tested phages exhibited consistent synergy with the bactericidal antibiotics gentamicin and fosfomycin. In contrast, combinations with the bacteriostatic agent nitrofurantoin produced predominantly neutral or antagonistic outcomes, with antagonism becoming more pronounced at low MOIs. This MOI dependence likely reflects the increased reliance of phage-mediated killing on intracellular replication at lower phage titers. At higher MOIs, elevated phage concentrations may partially offset the inhibitory effects of subinhibitory nitrofurantoin, preserving bacterial lysis. Nitrofurantoin disrupts ribosomal function and inhibits enzymes involved in DNA, RNA, and cell wall synthesis [43], thereby compromising host processes required for productive phage replication. Consistent with prior reports demonstrating antagonism between phages and bacteriostatic antibiotics such as linezolid and minocycline [42, 44], our findings suggest that suppression of bacterial metabolic activity can constrain phage amplification and alters infection kinetics, including reduced burst size and prolonged latent periods [45, 46], ultimately limiting therapeutic efficacy.

We also demonstrated that the temporal sequence of antimicrobial administration critically influences bacterial killing. Phage administration prior to gentamicin or fosfomycin resulted in greater lytic activity than simultaneous treatment, whereas reversing the order reduced efficacy. These results indicate that sequential regimens in which phages precede antibiotics are more effective than simultaneous, antibiotic-first or monotherapy approaches. Similar sequence-dependent effects have been reported for gentamicin combined with the *Pseudomonas* phage EPA1 [47, 48]. The enhanced efficacy of phage-first regimens may arise from multiple, nonexclusive mechanisms. The evolution of phage resistance can involve fitness trade-offs that increase bacterial susceptibility to antibiotics [49, 50], potentially sensitizing bacterial populations prior to antibiotic exposure. In addition, administering antibiotics after phage infection may avoid premature inhibition of phage replication, particularly for antibiotics such as gentamicin that suppress bacterial protein synthesis. Early phage amplification before antibiotic-mediated host killing may also increase effective phage abundance and contribute to improved bacterial clearance in phage-first regimens 10.1038/s44298-026-00222-4.

## Limitations

Despite the extensive insights gained from this project, associated limitations must be acknowledged. First, despite including multiple clinically relevant UPEC isolates, the scope of the study was limited with respect to the number of phages, bacteria, and antibiotics examined. Tripartite associations may therefore differ if alternative test panels are employed. Importantly, the inclusion of 25 UPEC isolates was not intended to provide exhaustive host range characterization, but to support the primary aim of this study, which was the evaluation of phage performance under physiologically relevant conditions and clinically relevant treatment regimens. Future studies incorporating larger and more diverse isolate collections will be necessary to further validate and generalize these findings. For phage stability and activity testing, urine from two female individuals was pooled. Given that urine composition can vary due to numerous physiological and environmental factors, sample provenance may influence experimental outcomes. Future studies using a broader range of urine samples and further characterization of urinary conditions will help to better understand the contribution of individual urine components to phage stability, activity, and phage–bacteria interactions. For assessing phage–antibiotic interactions, both phage and antibiotic concentrations were intentionally selected at relatively low, non-suppressive levels. This experimental design was chosen because complete growth suppression by either treatment alone would have precluded the detection of any additional benefit of the combination using optical density measurements. While we consider this approach a useful investigational method, it represents an important limitation with respect to clinical translation. In therapeutic settings, both phages and antibiotics are typically administered at concentrations intended to suppress or eradicate bacterial growth. Consequently, concentrations of phages and antibiotics at the site of infection would be expected to differ substantially from those used in our in vitro model. Likewise, the interval between administration of the two treatment modalities was selected to maximize the ability to detect differences in bacterial growth by optical density measurements. At the low concentrations employed in this study, administration of the second treatment at substantially later time points would likely not have produced a measurable effect, as bacterial growth would already have reached levels at which treatment efficacy is markedly reduced. Consequently, the timing intervals investigated here were chosen primarily to facilitate detection of treatment effects within the constraints of the experimental model. Although our findings suggest that administration sequence and timing may influence treatment efficacy, the optimal time lapse between phage and antibiotic administration in clinical settings remains uncertain and cannot be directly inferred from the present model. In vivo studies and clinical trials are required to determine whether the interactions observed under the experimental conditions used here are maintained under the higher and more complex exposure conditions encountered at the site of infection in patients.

An additional limitation of this study is the use of pooled urine derived from only two healthy donors. Although pooling was applied to reduce extreme inter-individual variability and to provide a standardized, physiologically relevant matrix, human urine exhibits substantial variability in pH, osmolarity, ionic strength, and metabolite composition. These factors may influence both bacterial growth behavior and phage infectivity, and therefore could affect the reproducibility and generalizability of the observed phage performance. Future studies should incorporate urine from a larger and more diverse donor cohort, as well as defined synthetic urine models, to systematically dissect the impact of urine heterogeneity on phage activity and to further validate the robustness of the observed effects.

Finally, the experimental systems of this study were designed primarily to evaluate phage stability, lytic activity, biofilm degradation, and phage–antibiotic interactions under physiologically relevant urine and bladder-like conditions. Although these models capture several environmental characteristics relevant to the urinary tract, they do not fully reproduce the complexity of rUTI pathophysiology, including host immune responses, urothelial invasion, intracellular bacterial reservoirs, tissue-specific pharmacokinetics, and patient-to-patient variability. Consequently, the present findings should be interpreted as providing insight into specific aspects of phage performance within the urinary tract environment rather than as a comprehensive model of rUTI.

## Conclusion

Recurrent urinary tract infections pose a major clinical challenge, driven by repetitive antimicrobial treatment, biofilm formation, and increasing antimicrobial resistance. Our comprehensive evaluation of six *E. coli* phages identified three promising candidates for therapeutic application. These phages demonstrated robust stability and strong lytic activity in urine, with performance largely preserved under physiologically relevant conditions, including variable pH and—depending on the phage—anaerobiosis. In silico depolymerase prediction reliably reflected in vitro biofilm-reducing capacity, supporting its use as an efficient pre-screening method. Synergistic interactions with gentamicin and fosfomycin, along with the superiority of phage-first administration, highlight the potential of rationally designed phage–antibiotic regimens. Finally, the quantitative frameworks introduced here enable objective comparison of phage performance under diverse conditions. Collectively, these findings provide a useful foundation for in vivo studies and clinical investigations aimed at optimizing phage-based approaches for rUTI.

### Summary points

- Identificatgion of phages G9062, G10400, and MM02 as suitable phages for the treatment of rUTI.
- The tested phages were stable under conditions mimicking the urinary tract, and in most cases, exhibited higher lytic activity in human urine than in phage buffer.
- Artificial alkalisation of urine to pH 7.5, as used in phage buffer, had variable effects on phage efficacy. Because the effect was minor and phage-dependent, general urine alkalisation cannot be recommended.
- Anaerobiosis affects phage lysis behaviour in a host-dependent manner.
- In silico depolymerase prediction correlated with in vitro biofilm reduction and is therefore a useful screening tool.
- Combination therapy with gentamicin or fosfomycin is favourable; however, the addition of nitrofurantoin should be avoided.
- The sequence of phage–antibiotic administration is crucial; phage application prior to antibiotics appears to be the superior strategy.
- A mathematical–numerical framework enabling objective assessment and comparative analysis of phage lysis behavior under different experimental conditions was developed.

## Materials and methods

### Bacterial strains

Uropathogenic *E. coli* isolates were provided by the Department of Medical Microbiology and Infection Control at the University Hospital Frankfurt. All bacteria were clinical urine isolates from patients with clinically confirmed UTI (≥ 100.000 CFU/mL with leukocyturia). The bacterial matrix included 25 diverse isolates, of which seven of which were identified as multidrug-resistant and/or ESBL-producing based on routine clinical microbiological diagnostics. This collection was selected to reflect clinically encountered heterogeneity in UPEC, particularly with respect to antimicrobial susceptibility profiles and patient population heterogeneity. Bacteria were aerobically grown at 37 °C in either Lysogeny Broth (LB; Roth, Germany) at 180 rpm or on LB 1,5% agar plates [51]. All isolates were stored in 25% glycerol stocks at -74 °C until required.

Anoxic culturing was done in prereduced Brain Heart Infustion (BHI) medium, flushed with 90% N_2_/ 10% CO_2_ and supplemented with 0.5 g/L L-cystein as a reducing agent, in serum bottles sealed with butyl rubber stoppers.

### Phage stocks

The six phages and their hosts used in this project were kindly provided and previously sequenced by the German Collection of Microorganisms and Cell Cultures (DSMZ) in Braunschweig and preselected in terms of host range on ESBL *E. coli*, as described earlier [15]. All phages belong to the class *Caudoviricetes* and display either myovirus (G10400, G2494, G9062, MM02, WFH) or podovirus (AC-L3) morphotypes. For practical reasons, a shortened form of the phage name (e.g., G10400 instead of vB_EcoM-G10400) is used throughout the text. Phage stocks were prepared by amplification on bacterial lawns using the double agar overlay method as previously described [16, 50, 52, 53]. Phages were propagated to produce webbed lysis plates, from which lysates were recovered using SM buffer. The lysates were centrifuged to remove bacterial and agar debris and subsequently sterilized by sequential filtration through 0.45 μm and 0.22 μm pore-size filters. Phage titers were determined by plaque assay. Stocks were stored at 4 °C. Phage AC-L3 (accession no. SRX15289991) was propagated on *E. coli* L3; phage G10400 (accession no. MK327937) on *E. coli* DSM 103,266; Phage G2494 (accession no. MK327935) on *E. coli* DSM 103,259; phage G9062 (accession no. MK373779) on *E. coli* DSM 103,265; Phage MM02 (accession no. MK373784) on *E. coli* DSM 498; phage WFH (accession no. MK373776) on *E. coli* DSM 101,139.

### Pooled human urine

Female urine from two individuals (one suffering from rUTI, one healthy woman) was centrifuged at 6,000 rpm for 25 min. The supernatant was consecutively passed through a 0.45 μm pore size filter. Sterility was confirmed by plating aliquots on LB agar. The pH value was measured and adapted where necessary for the experimental setup with potassium hydroxide or hydrochloric acid, followed by a final filtration step. Urine was stored at − 74 °C. Before use, samples were thawed and maintained at 4 °C for no longer than 48 h. The use of human urine samples, including a waiver of the requirement for informed consent to participate, was approved by the Institutional Review Board of Goethe University Frankfurt, Faculty of Medicine (ethics waiver no. 2023 − 1584).

### Double agar overlay method - MD/SP (Multiple Dilutions on Single Plates)

Host range and EOP were determined in biological and technical triplicates using the MD/SP (multiple dilutions on single plates) DAO method as previously described [16, 53, 54]. This spot assay-based method was used to assess phage infectivity and efficiency of plating across bacterial isolates. Overnight cultures were adjusted to an optical density at 600 nm (OD_600)_ of 0.1 and grown to an OD_600_ of approximately 0.2 before incorporation into 0.4% LB soft agar. Serial dilutions of phage stocks (2 µL) were spotted onto plates and incubated overnight at 37 °C. The appearance of spots was graded: CL (complete lysis); L + PRC (Lysis with phage resistant colonies); TL (turbid lysis); WP (web pattern); 0 (no visible plaque); xx (mean number where spots could be counted). Only when clear lysis and countable spots at the terminal dilution were apparent, the tested UPEC isolates were defined as susceptible.

### Efficiency of Plating (EOP)

The double agar overlay method was also used for EOP determination. Plaques on each plate at the final dilution were counted and the relative EOP was calculated as the ratio between the phage titer in PFU/mL for the test strain and the titer on the propagation host [55].

### Phage stability testing

For stability testing, phages with a titer of 10^9^ were diluted 1:10 in SM buffer (SM) or in human urine (U) and incubated over 24 h at 37 °C. Control phage stocks (C) were diluted 1:10 in SM buffer and stored at 4 °C. Subsequently, phages were serially diluted, and titers were determined on the respective host strain according to the above-described MD/SP method. Experiments were carried out in biological triplicates.

### Phage activity testing

Phage activity in SM buffer compared to pooled female urine was evaluated with planktonic phage activity assays as previously described [56]. Experiments were carried out using the Tecan Infinite M Plex Microplate Reader in U-shape 96 well plates (Merck, Germany). The assay was used to quantify phage-mediated bacterial growth inhibition in liquid culture under different conditions (SM buffer vs. urine). An overnight culture of the host strain was adjusted to an OD_600_ of 0.1 and grown for 1 h to an ∼OD_600_ of 0.2 (∼ 1 × 10^8^ CFU/mL) at 37 °C, 180 rpm. As bacterial growth proved to be insufficient in pure human urine in preliminary experiments (Supplementary Figue 7), 5% LB medium was added to ensure adequate nutrient availability. Accordingly, bacterial cultures were 1:10 diluted either in SM buffer or in human urine. Phage stocks were serially diluted in SM buffer or urine in 200-µL volumes. Bacteria and phages were combined in 96-well plates at defined MOIs (1, 0.01, 0.0001), resulting in a final matrix composition of 95% SM buffer or urine. OD_600_ readings were taken every 30 min for a total of 24 h with continuous shaking at 380 rpm. The microtiter plate had positive controls for every individual isolate (bacterial culture at an ∼OD_600_ of 0,2), as well as a negative control (LB only) and a blank (LB, Urine or SM buffer in a corresponding ratio). To evaluate to which extent the pH level is determinant for phage lysis behaviour in urine, in a sub-experiement, prior to testing, the urine pH was adjusted with potassium hydroxide to a value of 7.5 (as it is used in SM buffer). Each experiment was repeated in biological triplicates and the data was merged to give a single growth inhibition curve. Built on the premise that comparisons between the growth curves of phage infected and non-infected bacterial cultures can be used to quantitatively assess phage activity, virulence and AUC indices were determined (see also section: Virulence and AUC indices) [22]. The virulence index was calculated as the ratio of the area under the curve (AUC) of the bacterial reduction curve at a defined MOI against the phage-free control in either SM buffer or urine, substracted from 1. It quantifies lytic phage activity under a defined growth condition. To asses phage activity in urine compared to SM buffer, the AUC index was conceived: The value of the urine virulence index at a defined MOI was devided by the corresponding SM index at the same MOI and rated between 0.75 and 1.25 = phage as active in urine as in SM buffer; > 1.25 = higher phage activity in urine; < 0.75 = lower phage activity in urine.

### Phage activity under anoxic conditions

Activity of phages G9062 and MM02 under anoxic conditions was evaluated with PKAs. Brain Heart Infusion (BHI) medium was used because its composition supports metabolically active host cells even under oxygen-limited conditions, ensuring that observed differences in phage performance reflect the impact of oxygen availability rather than nutrient limitation. Briefly, anoxic overnight cultures of *E. coli* isolates UPEC 0310, UPEC 8457, UPEC 3603, and W3110 were subcultivated at 37 °C in prereduced BHI (Roth, Germany) in serum bottles sealed with butyl rubber stoppers until the cultures reached log phase (OD_600_ of 0.2–0.6). After adjusting the cultures to an OD_600_ of 0.2 with anoxic BHI, 100 µl each was transferred to a sterile 96-well clear flat-bottom plate (Greiner Bio-One, Germany) and mixed 1:1 with serial dilutions of the respective phages in anoxic BHI. Bacterial growth and lysis (OD_600_) at 37 °C was measured every 5 min in a plate reader (Tecan infinite 200Pro, Tecan, Switzerland)) for 18 h, shaking for 10 s before each measurement. The experiments were conducted as technical duplicates of three biological replicates. To maintain anoxic conditions throughout the experiment, handling of the isolates and phages was performed in an anaerobic chamber (Whitley DG250 Anaerobic Workstation, Don Whitley Scientific, UK) under a 98% N_2_/ 2% H_2_ (Linde Gas, Germany) gas atmosphere.

### In silico prediction of depolymerases

The previously sequenced genomes of the six *E. coli* phages were annotated using pharokka. To identify potential depolymerase enzymes, genomes were manually parsed for keywords related to depolymerases: “lysozyme,” “muramidase,” “hydrolase,” “sialidase,” “levanase,” “xylanase,” “dextranase,” “rhamnosidase,” “lyase,” “hyaluronidase,” “pectin,” “pectate,” “peptidase” and “lipase” [56–58]. Additionally, putative depolymerase genes were predicted with the machine learning based depolymerase finders PhageDPO [19] and DePP [20]. A threshold of 90% was chosen to identify a potential depolymerase gene. If a putative depolymerase gene was detected with a probablitiy of > 90% in one phage prediction tool whereas the probability was estimated < 50% in the second tool, the respective gene was not included. Directly neighbouring sequences likely belonging to the same enzyme were conflated as one hit. Following this methodology, a total of 38 sequences were identified as potentially encoding for phage depolymerases. Two of those hits were previously otherwise annotated by pharokka (e.g., lysis inhibitors) and were thus excluded as false positives.

### Biofilm reduction assays

Overnight cultures of six UPEC isolates grown in LB medium were diluted in human urine to achieve a final urine concentration of 90% (vol/vol) and an inoculum of 10⁸ CFU/mL. For biofilm establishment, 130 µL of each bacterial suspension were dispensed into the wells of an MBEC 96-peg-lid plate (Innovotech, Canada), including one negative control per isolate. Biofilms were grown for 48 h at 37 °C without shaking. Following incubation, planktonic cells were removed by immersing the peg lid in a PBS rinse plate for 10 s. The lid was then transferred to an infection plate prepared with phages at 10⁸ PFU/mL, diluted in 90% urine and matched to their respective bacterial hosts. Positive and negative control wells received an equal volume of sterile PBS. Plates were incubated for 12 h at 37 °C under static conditions. After phage exposure, a recovery plate was prepared by dispensing 130 µL of PBS into each well of a standard 96-well plate. The peg lid was rinsed twice in PBS, transferred to the recovery plate, and sonicated for 25 min to dislodge biofilms from the pegs, according to the manufacturer’s instructions. The recovered biofilm suspensions were serially diluted and spotted in duplicate onto square LB agar plates (1.5% agar) to determine viable CFU. Each phage–host pair was evaluated using four biological replicates and two technical replicates.

### Phage antibiotic interactions

The antimicrobial susceptibility of the isolates was determined using the Epsilometer Test (bioMérieux, Marcy l’Étoile, France) according to the manufacturer’s instructions and established clinical laboratory standards. Interactions between antibiotics and the three most promising phages for the treatment of UTI as determined in the preliminary experiments (G9062, G10400 and MM02) were investigated with a modification of the planktonic phage activity assay. An overnight culture of each host strain was adjusted to an OD₆₀₀ of 0.1 and incubated for 1 h at 37 °C with shaking (180 rpm) until reaching an OD₆₀₀ of ~ 0.2 (~ 1 × 10⁸ CFU/mL). Subsequently, 200 µL of the bacterial suspension in LB medium were transferred into a 96-well microtiter plate. Phages were added in 4 µL volumes of serial dilutions to achieve final MOIs of 0.1, 0.001, or 0.00001. Gentamicin, fosfomycin, or nitrofurantoin were added individually or in combination with phages at a final concentration corresponding to 0.25× MIC. Each plate included a positive control for every host strain, negative controls, and one blank. OD₆₀₀ measurements were recorded every 15 min for 48 h during continuous linear shaking at 380 rpm. Effects of phage–antibiotic combinations were assessed by monitoring reductions in optical density and quantified using virulence and area-under-the-curve (AUC) indices (Methods: Virulence and AUC indices).

In a subsequent experiment, we evaluated the optimal timing for the addition of antibiotics that had previously shown synergistic effects with phage G9062 (gentamicin and fosfomycin). Three treatment sequences were tested: administering phages and antibiotics simultaneously (SIM); phage application before antibiotics (PbA); administering phages after antibiotics (PaA). For simultaneous treatments, bacterial cultures adjusted to OD₆₀₀ ≈ 0.2 in LB medium received both phage (MOI 0.001) and one of the selected antibiotics at 0.25 × MIC in 4 µL volumes and were incubated for 24 h. For PbA treatments, phage was added first and incubated for 4 h before adding the antibiotic, followed by an additional 20 h of incubation. In the reverse sequential combination (PaA), antibiotic was added 4 h prior to phage. To verify whether observed clearing corresponded to bactericidal activity, bacterial enumeration was performed after 24 h. Samples from each well at the endpoint of the experiment were serially diluted in LB medium. For CFU determination, 10 µL of each dilution were spotted onto LB agar plates (1.5%), allowed to dry, and incubated overnight at 37 °C in an inverted position.

### Statistical analysis

R (Version 4.5.1) [59] was used for data collection, statistical computing, and visualization. Phage titers were compared between conditions using Kruskal–Wallis tests, and titer ratios were compared using two-sided Mann–Whitney–Wilcoxon tests. EOP values were compared between phages across different UPEC isolates using Kruskal–Wallis tests. VI, V_i_(t)_max_, and t_max_ were compared across different MOI using Kruskal–Wallis or two-sided Mann–Whitney–Wilcoxon tests, depending on the number of groups analyzed. AUCI were compared to unity across different MOI using Wilcoxon signed-rank tests. Bacterial counts, measured as CFU/mL, were compared using Kruskal–Wallis or two-sided Mann–Whitney–Wilcoxon tests, as appropriate. For Mann-Whitney-Wilcoxon and Wilcoxon signed rank tests exact p-values were computed using the coin R package (version 1.4-3).

A significance level of 0.05 was applied throughout. P-values were adjusted for multiple testing per outcome using the Holm–Bonferroni method, except for analyses assessing phage stability, to minimize the risk of Type II errors. Dunn’s tests were used for post hoc pairwise comparisons following Kruskal–Wallis tests that remained significant after multiple testing correction. Post hoc p-values were independently adjusted for multiple comparisons within each test.

### Virulence and AUC indices

For this study, an objective methodology linking experimental data with mathematical models to objectively characterize phage lysis dynamics was established based on the previously described virulence index [22]. In contrast to the original VI, which quantifies phage virulence under a single experimental condition, the framework enables comparative assessment of phage efficacy across different environmental conditions and treatment regimens. Growth curves of infected and non-infected bacterial cultures were used to assess the virulence of phages (p) under defined environmental conditions (e.g., urine, anaerobiosis, antibiotics). The integrated area under the bacterial reduction curve (AP_10_^x^, where 10^x^ is the MOI) was compared with the integrated area under the phage-free control (A0) from the onset of infection to the endpoint of the experiment.

• Virulence Index (VI):

The virulence index was calculated from the AUC of the bacterial reduction curve at a defined MOI, divided by the AUC of the respective curve of the growth control, and subtracted from 1. It quantifies phage activity at a defined MOI (10ˣ) and growth condition (C₁) relative to the corresponding phage-free control (A0). Higher values indicate stronger overall phage activity over the measured time period.

$$\mathrm{VI}_{\mathrm{c}1}=1-\mathrm{A}_{\mathrm{c}1,10^{\mathrm{x}}}/\;\mathrm{A}0_{\mathrm{c}1}$$

- Temporal Phage Virulence (VI(t)):

Like the virulence index, the temporal phage virulence VI(t)) was determined by dividing the OD_600_ of the lysis curves of phage-treated samples by the OD_600_ of the growth control at every time point of the measurement, eventually subtracting these ratios from 1, where VI(t) = 1 equals full growth inhibition and VI(t) = 0 equals no growth inhibition at each time point. Plotted against time, these temporal virulence profiles are used to determine the time point of maximum virulence (t_max_) and the corresponding maximum virulence value (VI(t)_max_).

- AUC Index (AUCI):

The AUC index compares phage activity at the same MOI (10ˣ) across two different growth conditions (C₁ and C₂). Higher values indicate greater phage activity under condition C₁ relative to C₂.

$$\mathrm{AUCI}_{\left(\mathrm{C1}/\mathrm{c2}\right),10^{\mathrm{x}}}=\mathrm{VI}_{\mathrm{c1,10}^{\mathrm{x}}}/\mathrm{VI}_{\mathrm{c2},10^{\mathrm{x}}}$$

### Language optimization

The text was edited to improve clarity and refine the English language with the assistance of generative AI tools, specifically ChatGPT (GPT-4.0 and GPT-5.0). Nevertheless, the proposal was revised, reviewed, and finalized by the authors, who take full responsibility for its content.

## Supplementary Information

Supplementary Material 1.

## Data Availability

All other data supporting the findings of this study are available within the article, its Supplementary information files, and from the corresponding author upon reasonable request.
