# Supplementary material for: Evaluation of a phage-inclusive multimodal strategy for recurrent urinary tract infections
Source: BMC Microbiol. 2026 Aug 12;26:732. doi: 10.1186/s12866-026-05524-4 (PMC13474533; doi:10.1186/s12866-026-05524-4)
Supplement: Supplementary file 1 [file 12866_2026_5524_MOESM1_ESM.docx]

**Supplementary data**

**Supplementary Figure 1: Host range.** Host range and box plot representation of the EOP values across the tested bacterial panel (uropathogenic E. coli, n=25). Color-coded dots represent individual UPEC isolates.  Isolates 3382, 8993, 7900, and 8484 are omitted, as they were resistant to all tested phages. Host range: phage G10400 = 28%; phage G9062 = 48%; phage G2494 = 24%; phage AC-L3 32%; phage MM02 = 28%; phage WFH = 24%. Median EOP values: phage G10400 = 0.3; phage G9062 = 1.5; phage G2494 = 2.55; phage AC-L3 0.06; phage MM02 = 0.08; phage WFH = 0.17. The Kruskal-Wallis test, followed by Dunn’s test yielded P values of 0.01.

**Supplementary Figure 2: Phage lysis behaviour in SM buffer and urine**. Bacterial reduction curves for phages AC-L3, G10400, G2494, G9062, MM02, and WFH with their respective propagation hosts in SM buffer, urine pH 6.3 (native), and urine pH 7.5 (after artificial pH increment).

**Supplementary Figure 3: Comparative overview of phage virulence under oxic and anoxic conditions.** Bacterial reduction curves for phages G9062 and MM02 with UPEC 0310, UPEC 8457, UPEC 3603 and *E. coli* W3110 under aerobic or anaerobic growth conditions.

| **Phage** | **Phage DPO**  **(cut off 90%) (18)** | **DePP (19)**  **(cut off 90%)** | **Manual parsing**  **(34; 36)** |
| --- | --- | --- | --- |
| L3 | ~~NODE_1_length_77016_cov_47.857460_CDS_0009 hypothetical protein (97%)~~  NODE_1_length_77016_cov_47.857460_CDS_0141 tail fiber protein; host specificity (99%) (36)  NODE_1_length_77016_cov_47.857460_CDS_0145 tail fiber protein (84%)  NODE_1_length_77016_cov_47.857460_CDS_0146 tail fiber protein (97%) | ~~NODE_1_length_77016_cov_47.857460_CDS_0009 (48%)~~  NODE_1_length_77016_cov_47.857460_CDS_0141(96%)  NODE_1_length_77016_cov_47.857460_CDS_0145 (93%)  NODE_1_length_77016_cov_47.857460_CDS_0146 (95%) | ~~-~~ |
| G10400 | SEXZKHDG_CDS_0160 baseplate wedge subunit (92%)  SEXZKHDG_CDS_0164 baseplate wedge subunit (98%)  SEXZKHDG_CDS_0166 tail collar fiber protein (96%)  ~~SEXZKHDG_CDS_0168 head-tail adaptor Ad2 (90%)~~  SEXZKHDG_CDS_0173 tail sheath (99%)  ~~SEXZKHDG_CDS_0261 tail fiber protein proximal subunit (44%)~~  SEXZKHDG_CDS_0263 hinge connector of long tail fiber protein distal connector (92%)  SEXZKHDG_CDS_0264 long tail fiber protein distal subunit (99%)  ~~SEXZKHDG_CDS_0288 RIIB lysis inhibitor (90%)~~ (false positive) | SEXZKHDG_CDS_0160 (93%)  SEXZKHDG_CDS_0161 (94%)  SEXZKHDG_CDS_0164 (92%)  SEXZKHDG_CDS_0166 (86%)  ~~SEXZKHDG_CDS_0168 (49%)~~  SEXZKHDG_CDS_0173 (92%)  ~~SEXZKHDG_CDS_0261 (97%)~~  SEXZKHDG_CDS_0263 (73%)  SEXZKHDG_CDS_0264 (96%) | SEXZKHDG_CDS_0157 baseplate hub subunit and tail lysozyme  SEXZKHDG_CDS_0131 nudix hydrolase |
| G2494 | ~~YQXEOMEP_CDS_0149 baseplate wedge tail fiber protein connector (95%)~~  YQXEOMEP_CDS_0150 baseplate wedge subunit (95%)  YQXEOMEP_CDS_0153 fibritin neck whisker (94%)  ~~YQXEOMEP_CDS_0154 head-tail adaptor Ad2 (93%)~~  YQXEOMEP_CDS_0162 tail sheath (96%)  YQXEOMEP_CDS_0269 tail fiber protein proximal subunit (80%)  YQXEOMEP_CDS_0271 tail fiber protein (97%)  YQXEOMEP_CDS_0272 long tail fiber protein distal subunit (91%) | ~~YQXEOMEP_CDS_0149 (44%)~~  YQXEOMEP_CDS_0150 (87%)  YQXEOMEP_CDS_0153 (66%)  ~~YQXEOMEP_CDS_0154 (30%)~~  YQXEOMEP_CDS_0162 (95%)  YQXEOMEP_CDS_0269 (95%)  YQXEOMEP_CDS_0271 (79%)  YQXEOMEP_CDS_0272 (95%) | YQXEOMEP_CDS_0138 baseplate hub subunit and tail lysozyme |
| G9062 | TCUKYVRS_CDS_0165 baseplate wedge subunit (92%)  ~~TCUKYVRS_CDS_0166 baseplate wedge subunit (26%)~~  TCUKYVRS_CDS_0169 baseplate wedge subunit (98%)  TCUKYVRS_CDS_0171 tail collar fiber protein (97%)  TCUKYVRS_CDS_0173 head-tail adaptor Ad2 (91%)  TCUKYVRS_CDS_0181 tail sheath (99%)  TCUKYVRS_CDS_0262 tail fiber protein proximal subunit (50%)  TCUKYVRS_CDS_0265 long tail fiber protein distal subunit (98%)  ~~TCUKYVRS_CDS_0290 RIIB lysis inhibitor (91%)~~ (false positive) | TCUKYVRS_CDS_0165 (92%)  ~~TCUKYVRS_CDS_0166 (94%)~~  TCUKYVRS_CDS_0169 (93%)  TCUKYVRS_CDS_0171 (86%)  TCUKYVRS_CDS_0173 (51%)  TCUKYVRS_CDS_0181 (94%)  TCUKYVRS_CDS_0262 (96%)  TCUKYVRS_CDS_0265 (95%) | TCUKYVRS_CDS_0136 nudix hydrolase  TCUKYVRS_CDS_0162 baseplate hub subunit and tail lysozyme |
| MM02 | PGMCPWJY_CDS_0046 peptidase (92%)  PGMCPWJY_CDS_0165 baseplate wedge subunit (89%)  ~~PGMCPWJY_CDS_0166 baseplate wedge subunit (28%)~~  PGMCPWJY_CDS_0169 baseplate wedge subunit (97%)  PGMCPWJY_CDS_0171 tail collar fiber protein (99%)  PGMCPWJY_CDS_0172 fibritin neck whisker (99%)  PGMCPWJY_CDS_0178 tail sheath (100%)  PGMCPWJY_CDS_0255 tail fiber protein proximal subunit (75%)  PGMCPWJY_CDS_0258 long tail fiber protein distal subunit (97%)  Total of 10 potential depolymerase genes | PGMCPWJY_CDS_0046 (57%)  PGMCPWJY_CDS_0165 (92%)  ~~PGMCPWJY_CDS_0166 (93%)~~  PGMCPWJY_CDS_0169 (92%)  PGMCPWJY_CDS_0171 (81%)  PGMCPWJY_CDS_0172 (56%)  PGMCPWJY_CDS_0178 (93%)  PGMCPWJY_CDS_0255 (96%)  PGMCPWJY_CDS_0258 (93%) | PGMCPWJY_CDS_0046 peptidase  PGMCPWJY_CDS_0162 baseplate hub subunit and tail lysozyme  PGMCPWJY_CDS_0128 nudix hydrolase |
| WFH | XJDQSGKP_CDS_0018 tail fiber protein (97%)  XJDQSGKP_CDS_0057 hypothetical protein (96%)  XJDQSGKP_CDS_0058 tail protein (94%) | XJDQSGKP_CDS_0018 (97%)  XJDQSGKP_CDS_0057 (96%)  XJDQSGKP_CDS_0058 (91%) | - |

**Supplementary table 1: Putative depolymerase genes.** Putative depolymerase genes predicted with phage DPO, DePP and manual parsing (“lysozyme”, “muramidase”, “hydrolase”, “sialidase”, “levanase”, “xylanase”, “dextranase”, “rhamnosidase”, “lyase”, “hyaluronidase”, “pectin”, “pectate”, “peptidase” and “lipase”). The phages MM02 (n = 10) and G9062 (n= 9) were found to have the most putative depolymerase genes. Crossed out genes ~~(xxx)~~ were eliminated if a putative depolymerase gene was detected with a probablitiy of > 90% in one phage prediction tool when the probability was estimated < 50% in the second tool. As directly neighbouring sequences with a depolymerase probability of > 90% most likely belong to the same enzyme they were conflated as one hit (marked in yellow).

| **Phage** | **In vitro** (biofilm reduction in %) | **In silico prediction** (number of putative depolymerase genes) | | | | **Halo expression** (diameter in mm) |
| --- | --- | --- | --- | --- | --- | --- |
|  |  | **Total** | **DPO** | **DePP** | **Manual**  **parsing** |  |
| MM02 | 99.998 | 10 | 5 | 6 | 3 | 2,5 |
| G9062 | 99.8 | 9 | 6 | 6 | 2 | 2 |
| G2494 | 99 | 6 | 6 | 3 | 1 | 2 |
| WFH | 97 | 3 | 2 | 2 | 0 | - |
| Ac-L3 | 95 | 2 | 3 | 2 | 0 | - |
| G10400 | 37 | 7 | 6 | 5 | 2 | 1 |

**Supplementary table 2: Comparative table of in silico prediction and halo expression with in vitro biofilm reduction.** Overall, depolymerase prediction tools and halo formation correlated well with in vitro biofilm degradation.

| **Antibiotics (resistant break point, mg/L)** | **Strain** | **MIC (mg/L)** |
| --- | --- | --- |
| Fosfomycin (8) | *E. coli* DSM 103265 | 0,5 |
|  | *E. coli* DSM 498 | 0,5 |
|  | *E. coli* DSM 03266 | 0,5 |
| Gentamicin (2) | *E. coli* DSM 103265 | 1 |
|  | *E. coli* DSM 498 | 0,5 |
|  | *E. coli* DSM 03266 | 1 |
| Nitrofurantoin (64) | *E. coli* DSM 103265 | 16 |
|  | *E. coli* DSM 498 | 8 |
|  | *E. coli* DSM 03266 | 16 |

**Supplementary table 3: Minimal inhibitory concentrations (MIC).** Minimal inhibitory concentrations for propagation hosts UPEC DSM 103465 (for phage G9062), UPEC DSM 498 (for phage MM02) and UPEC DSM 03266 (for phage G10400). MIC breakpoints are based on the EUCAST 2024 clinical MIC breakpoints for enterobacterales.

**Supplementary Figure 4: Phage antibiotic interactions.** Bacterial reduction curves for fosfomycin, gentamicin or nitrofurantoin at 0.25 MIC with phages G9062, G10400 or MM02 on their propagation hosts at MOIs ranging from 10^-1,^ 10^-3^ to 10^-5^. Abbreviations: PC, positive control.

**Supplementary Figure 5: Phage antiobiotic treatment sequence.** Bacterial reduction curves for different treatment schedules of fosfomycin or gentamicin at 0.25 x MIC with phage G9062 on its propagation host DSM 103265: simultaneous combination, phage addition 4 h before antibiotics, and phage addition 4 h after antibiotics. Abbreviations: PC, positive control; FOS, fosfomycin; GEN, gentamicin.

**Supplementary Figure 6: Bacterial survival after 24h exposure to phage antibiotic combination sequences.** Fosfomycin or gentamicin at 0.25 MIC were combined with phage G9062 on its host DSM 103465 at an MOI of 10-3 in different treatment sequences: administration of phages and antibiotics simultaneously, phage application 4 hours before antibiotics, phage application 4 hours after antibiotics. Initial bacterial counts were 10^8^ CFU/mL. After 24 hours, bacterial enumeration tests were performed. Asterisks denote statistical significance calculated across all three MOIs. The Kruskal-Wallis test, followed by Dunn’s test yielded P values of < 0.05 (*).

**Supplementary Figure 7: Bacterial growth and phage inhibition at varying LB supplementation levels in urine.** To determine the minimum LB supplementation required for phage activity testing in urine, UPEC strains 3947, 7958, and 8923 were cultured in urine supplemented with different concentrations of LB medium (5%, 10%, or 50% v/v). These conditions were compared with LB medium alone and urine alone. Bacterial growth was assessed in the presence or absence of the corresponding phages G9062, G10400, and MM02, respectively. Growth kinetics are shown in **(A),** and the corresponding AUC values are presented in **(B)**. Abbreviations: AUC, area under the curve.

**Supplementary Figure 8:** Table summarizing the origin, antibiotic susceptibility profiles, and experimental use of all *E. coli* isolates included in this study.

*Phage susceptibility was determined using the DAO assay.

Abbreviations: ESBL, extended-spectrum β-lactamase producer; 3 MRGN, Gram-negative bacteria resistant to three of four key antibiotic classes used for treatment (German classification for multidrug-resistant Gram-negative bacteria); UHF, University Hospital Frankfurt; UHB, University Hospital Bonn; DSMZ, German Collection of Microorganisms and Cell Cultures.
